# Supplementary material for: High Molecular Weight Fucoidan Restores Intestinal Integrity by Regulating Inflammation and Tight Junction Loss Induced by Methylglyoxal-Derived Hydroimidazolone-1
Source: Mar Drugs. 2022 Sep 17;20(9):580. doi: 10.3390/md20090580 (PMC9505531; doi:10.3390/md20090580)
Supplement: Supplementary file 1 [file marinedrugs-20-00580-s001.zip › marinedrugs-1840342-supplementary.pdf]

marine drugs

*Supplementary Material for*

# High molecular weight fucoidan restores intestinal integrity by regulating inflammation and tight junction loss induced by methylglyoxal-derived hydroimidazolone-1

**Jae-Min Lim [1], Hee Joon Yoo [1] and Kwang-Won Lee [1,2*]**

1 Department of Biotechnology, College of Life Science and Biotechnology, Korea University, Seoul 02841, Korea
2 Department of Food Bioscience and Technology, College of Life Science and Biotechnology, Korea University, Seoul 02841, Republic of Korea
* Correspondence: kwangwon@korea.ac.kr; Tel.: +82-2-3290-3027

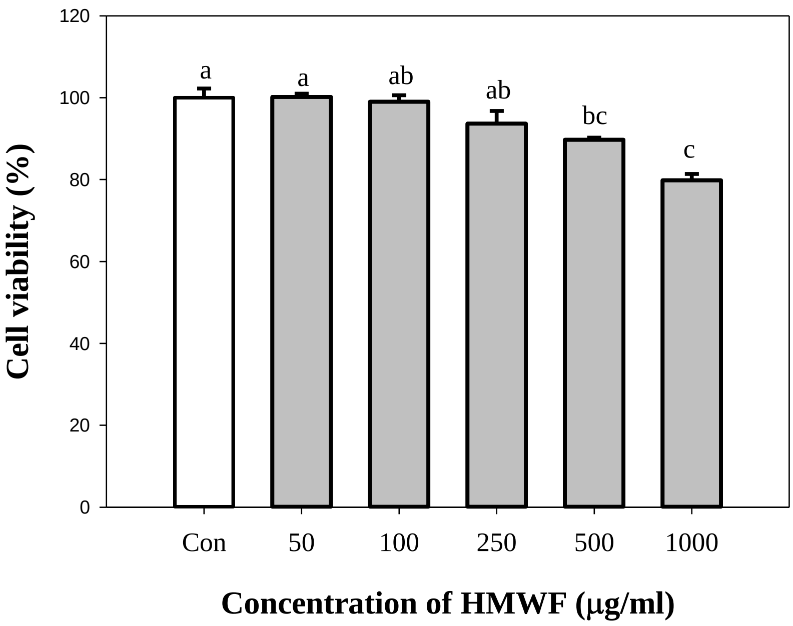


**Figure S1**. Effect of HMWF on cell viability of Caco-2 cells. Cells were treated with 0 to 1,000 μg/mL HMWF for 24 h. Caco-2 cell viability was evaluated by MTT assay. The results are shown as mean ± standard deviation (SD) (n=3). [a-c] Different superscripts mean significant difference by Tukey's multiple range test ($p < 0.05$).

**Table S1**. Quantitative reverse transcription polymerase chain reaction (qRT-PCR) primers.

| Origin | Marker | | Sequence (5'→3') |
|---|---|---|---|
| Human | ZO-1 | Forward | TTC ACG CAG TTA CGA GCA AG |
| | | Reverse | TTG GTG TTT GAA GGC AGA GC |
| | Occludin | Forward | GGG CAT TGC TCA TCC TGA AG |
| | | Reverse | GCC TGT AAG GAG GTG GAC TT |
| | Claudin-1 | Forward | TGG TCA GGC TCT CTT CAC TG |
| | | Reverse | TTG GAT AGG GCC TTG GTG TT |
| | RAGE | Forward | CTA CCG AGT CCG TGT CTA CCA |
| | | Reverse | CAT CCA AGT GCC AGC TAA GAG |
| | TNF-$\alpha$ | Forward | GTC AGA TCA TCT TCT CGA ACC |
| | | Reverse | CAG ATA GAT GGG CTC ATA CC |
| | IL-6 | Forward | GAC AGC CAC TCA CCT CTT CA |
| | | Reverse | GAC AGC CAC TCA CCT CTT CA |
| | GAPDH | Forward | TGC ACC ACC AAC TGC TTA GC |
| | | Reverse | GGC ATG GAC TGT GGT CAT GAG |
| Mouse | ZO-1 | Forward | AGA AGA TAG CCC TGC AGC |
| | | Reverse | AGT CCG TAA GGA GAT TCT |
| | Occludin | Forward | GGT CA GGG AAT ATC CAC C |
| | | Reverse | ATT ATA TTC ATC AGC AGC |
| | Claudin-1 | Forward | CAA CGC GGG GCT GCA GCT |
| | | Reverse | TTG TTT TCC GGG GAC AGG A |
| | RAGE | Forward | CTG GCA CTT AGA TGG GAA ACT |
| | | Reverse | GCT CTG ACC GCA GTG TAA A |
| | TNF-$\alpha$ | Forward | TAG CCA GGA GGG AGA ACA GA |
| | | Reverse | TTT TCT GGA GGG AGA TAT GG |
| | IL-6 | Forward | GAC AAC CAC GGC CTT CCC TA |
| | | Reverse | ATC TTT TGG GGT CCG TCA ACT |
| | GAPDH | Forward | CAT GGC CTT CCG TGT TC |
| | | Reverse | CCT GGT CCT CAG TGT AGC |
